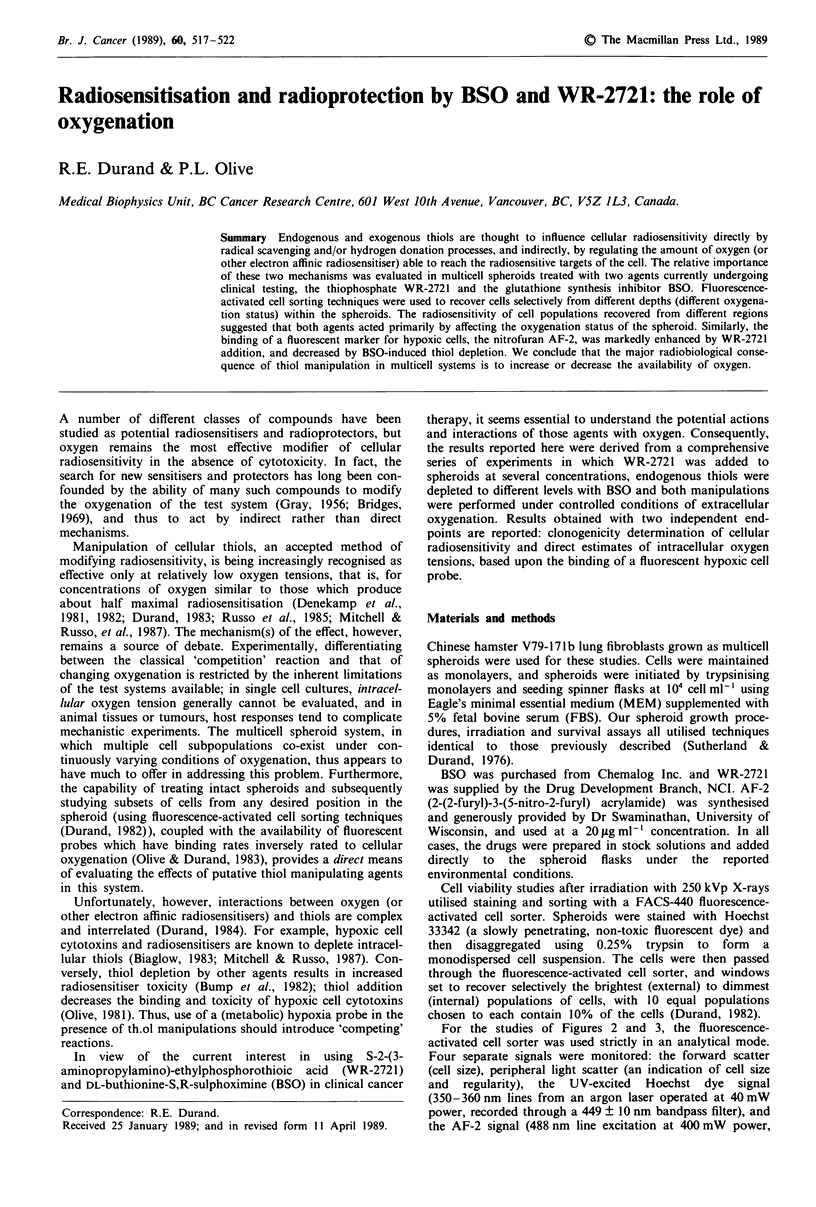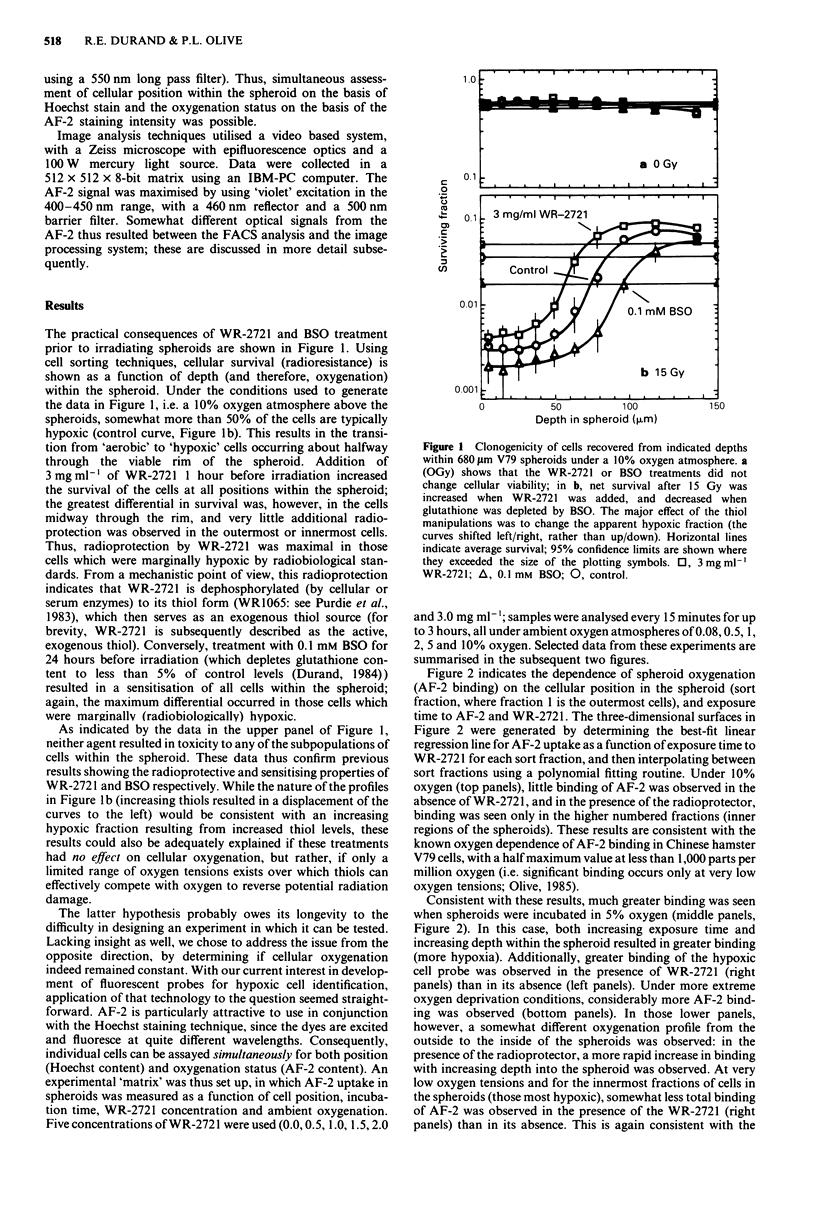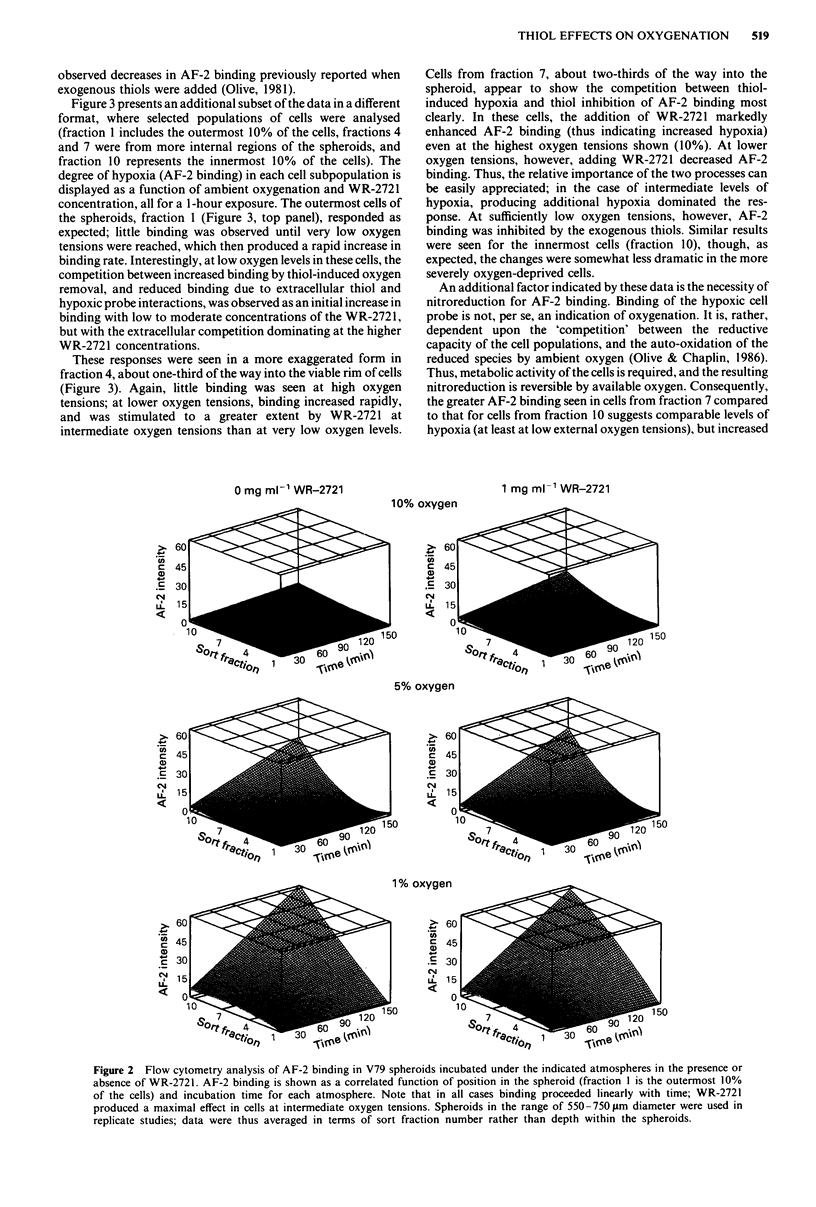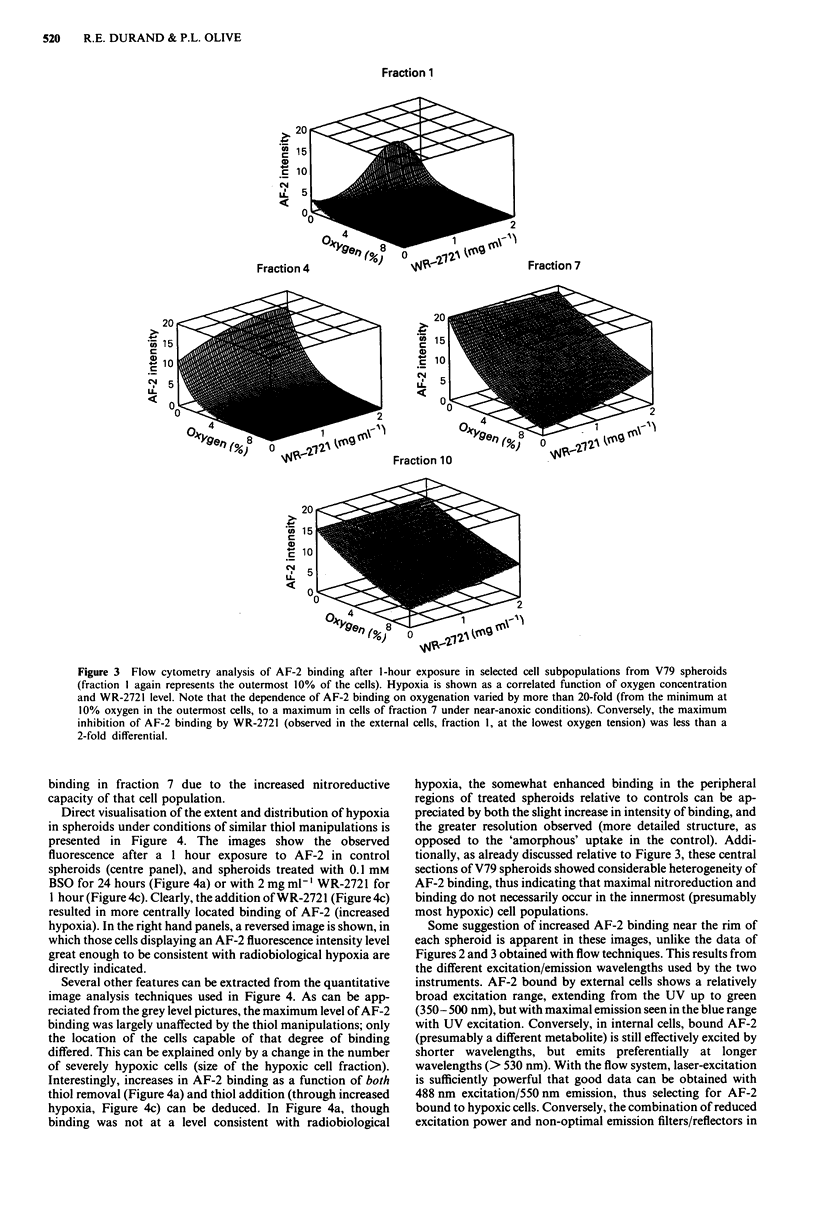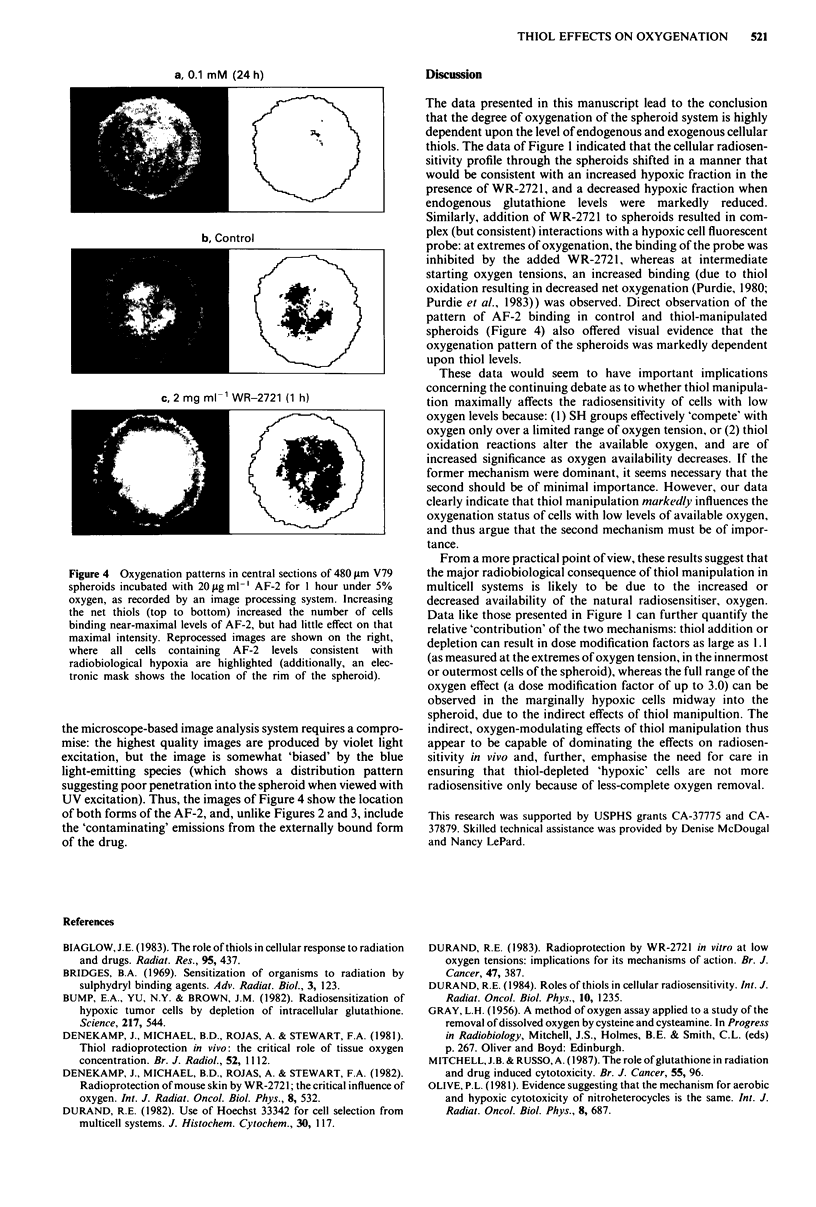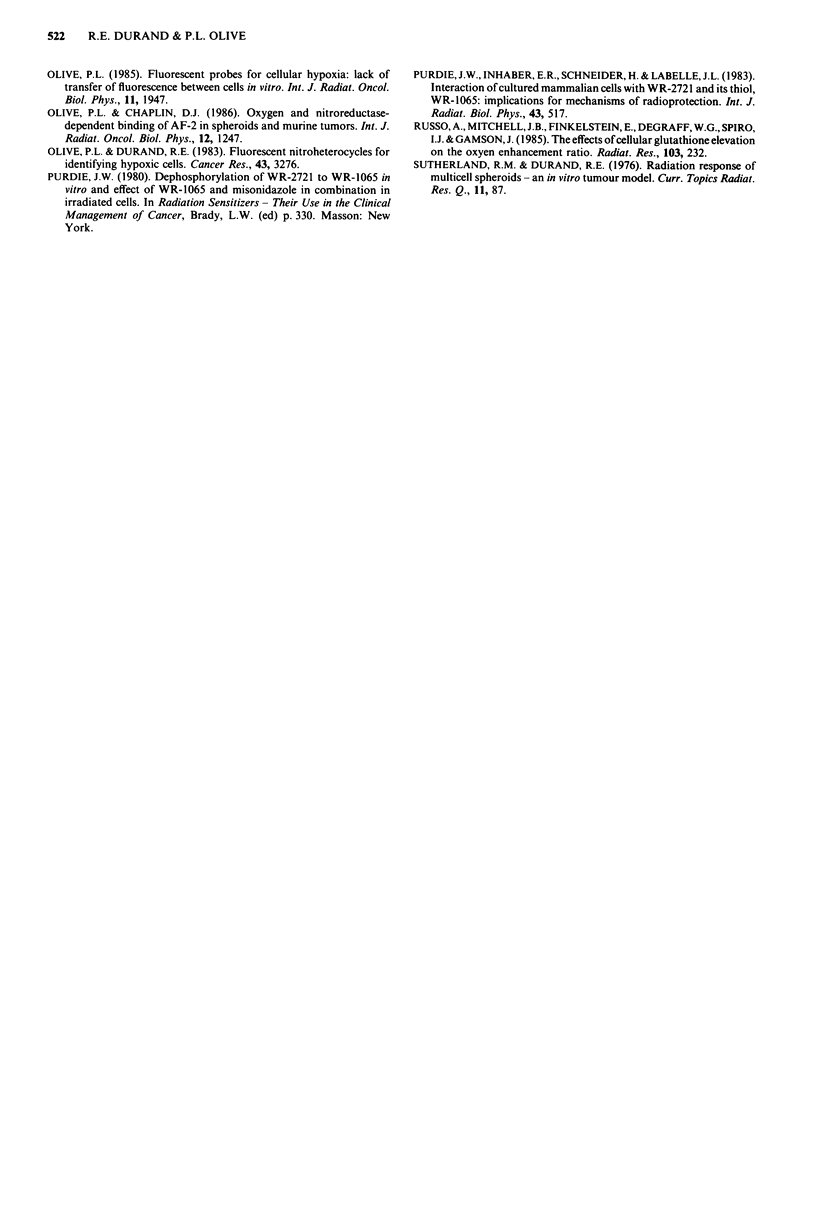# Radiosensitisation and radioprotection by BSO and WR-2721: the role of oxygenation.

**DOI:** 10.1038/bjc.1989.305

**Published:** 1989-10

**Authors:** R. E. Durand, P. L. Olive

**Affiliations:** Medical Biophysics Unit, BC Cancer Research Centre, Vancouver, Canada.

## Abstract

**Images:**


Br. J. Cancer (1989), 60, 517–522 © The Macmillan Press Ltd., 1989

### Radiosensitisation and radioprotection by BSO and WR-2721: the role of oxygenation

R.E. Durand & P.L. Olive

*Medical Biophysics Unit, BC Cancer Research Centre, 601 West 10th Avenue, Vancouver, BC, V5Z 1L3, Canada.*

**Summary** Endogenous and exogenous thiols are thought to influence cellular radiosensitivity directly by radical scavenging and/or hydrogen donation processes, and indirectly, by regulating the amount of oxygen (or other electron affinic radiosensitiser) able to reach the radiosensitive targets of the cell. The relative importance of these two mechanisms was evaluated in multicell spheroids treated with two agents currently undergoing clinical testing, the thiophosphate WR-2721 and the glutathione synthesis inhibitor BSO. Fluorescence-activated cell sorting techniques were used to recover cells selectively from different depths (different oxygenation status) within the spheroids. The radiosensitivity of cell populations recovered from different regions suggested that both agents acted primarily by affecting the oxygenation status of the spheroid. Similarly, the binding of a fluorescent marker for hypoxic cells, the nitrofuran AF-2, was markedly enhanced by WR-2721 addition, and decreased by BSO-induced thiol depletion. We conclude that the major radiobiological consequence of thiol manipulation in multicell systems is to increase or decrease the availability of oxygen.

A number of different classes of compounds have been studied as potential radiosensitisers and radioprotectors, but oxygen remains the most effective modifier of cellular radiosensitivity in the absence of cytotoxicity. In fact, the search for new sensitisers and protectors has long been confounded by the ability of many such compounds to modify the oxygenation of the test system (Gray, 1956; Bridges, 1969), and thus to act by indirect rather than direct mechanisms.

Manipulation of cellular thiols, an accepted method of modifying radiosensitivity, is being increasingly recognised as effective only at relatively low oxygen tensions, that is, for concentrations of oxygen similar to those which produce about half maximal radiosensitisation (Denekamp *et al.*, 1981, 1982; Durand, 1983; Russo *et al.*, 1985; Mitchell & Russo, *et al.*, 1987). The mechanism(s) of the effect, however, remains a source of debate. Experimentally, differentiating between the classical 'competition' reaction and that of changing oxygenation is restricted by the inherent limitations of the test systems available; in single cell cultures, *intracellular* oxygen tension generally cannot be evaluated, and in animal tissues or tumours, host responses tend to complicate mechanistic experiments. The multicell spheroid system, in which multiple cell subpopulations co-exist under continuously varying conditions of oxygenation, thus appears to have much to offer in addressing this problem. Furthermore, the capability of treating intact spheroids and subsequently studying subsets of cells from any desired position in the spheroid (using fluorescence-activated cell sorting techniques (Durand, 1982)), coupled with the availability of fluorescent probes which have binding rates inversely rated to cellular oxygenation (Olive & Durand, 1983), provides a *direct* means of evaluating the effects of putative thiol manipulating agents in this system.

Unfortunately, however, interactions between oxygen (or other electron affinic radiosensitisers) and thiols are complex and interrelated (Durand, 1984). For example, hypoxic cell cytotoxins and radiosensitisers are known to deplete intracellular thiols (Biaglow, 1983; Mitchell & Russo, 1987). Conversely, thiol depletion by other agents results in increased radiosensitiser toxicity (Bump *et al.*, 1982); thiol addition decreases the binding and toxicity of hypoxic cell cytotoxins (Olive, 1981). Thus, use of a (metabolic) hypoxia probe in the presence of th.ol manipulations should introduce 'competing' reactions.

In view of the current interest in using S-2-(3-aminopropylamino)-ethylphosphorothioic acid (WR-2721) and DL-buthionine-S,R-sulphoximine (BSO) in clinical cancer therapy, it seems essential to understand the potential actions and interactions of those agents with oxygen. Consequently, the results reported here were derived from a comprehensive series of experiments in which WR-2721 was added to spheroids at several concentrations, endogenous thiols were depleted to different levels with BSO and both manipulations were performed under controlled conditions of extracellular oxygenation. Results obtained with two independent endpoints are reported: clonogenicity determination of cellular radiosensitivity and direct estimates of intracellular oxygen tensions, based upon the binding of a fluorescent hypoxic cell probe.

### Materials and methods

Chinese hamster V79-171b lung fibroblasts grown as multicell spheroids were used for these studies. Cells were maintained as monolayers, and spheroids were initiated by trypsinising monolayers and seeding spinner flasks at $10^4$ cell $ml^{-1}$ using Eagle's minimal essential medium (MEM) supplemented with 5% fetal bovine serum (FBS). Our spheroid growth procedures, irradiation and survival assays all utilised techniques identical to those previously described (Sutherland & Durand, 1976).

BSO was purchased from Chemalog Inc. and WR-2721 was supplied by the Drug Development Branch, NCI. AF-2 (2-(2-furyl)-3-(5-nitro-2-furyl) acrylamide) was synthesised and generously provided by Dr Swaminathan, University of Wisconsin, and used at a 20 $\mu g\ ml^{-1}$ concentration. In all cases, the drugs were prepared in stock solutions and added directly to the spheroid flasks under the reported environmental conditions.

Cell viability studies after irradiation with 250 kVp X-rays utilised staining and sorting with a FACS-440 fluorescence-activated cell sorter. Spheroids were stained with Hoechst 33342 (a slowly penetrating, non-toxic fluorescent dye) and then disaggregated using 0.25% trypsin to form a monodispersed cell suspension. The cells were then passed through the fluorescence-activated cell sorter, and windows set to recover selectively the brightest (external) to dimmest (internal) populations of cells, with 10 equal populations chosen to each contain 10% of the cells (Durand, 1982).

For the studies of Figures 2 and 3, the fluorescence-activated cell sorter was used strictly in an analytical mode. Four separate signals were monitored: the forward scatter (cell size), peripheral light scatter (an indication of cell size and regularity), the UV-excited Hoechst dye signal (350–360 nm lines from an argon laser operated at 40 mW power, recorded through a 449 ± 10 nm bandpass filter), and the AF-2 signal (488 nm line excitation at 400 mW power,

Correspondence: R.E. Durand.
Received 25 January 1989; and in revised form 11 April 1989.

using a 550 nm long pass filter). Thus, simultaneous assessment of cellular position within the spheroid on the basis of Hoechst stain and the oxygenation status on the basis of the AF-2 staining intensity was possible.

Image analysis techniques utilised a video based system, with a Zeiss microscope with epifluorescence optics and a 100 W mercury light source. Data were collected in a 512 × 512 × 8-bit matrix using an IBM-PC computer. The AF-2 signal was maximised by using 'violet' excitation in the 400–450 nm range, with a 460 nm reflector and a 500 nm barrier filter. Somewhat different optical signals from the AF-2 thus resulted between the FACS analysis and the image processing system; these are discussed in more detail subsequently.

### Results

The practical consequences of WR-2721 and BSO treatment prior to irradiating spheroids are shown in Figure 1. Using cell sorting techniques, cellular survival (radioresistance) is shown as a function of depth (and therefore, oxygenation) within the spheroid. Under the conditions used to generate the data in Figure 1, i.e. a 10% oxygen atmosphere above the spheroids, somewhat more than 50% of the cells are typically hypoxic (control curve, Figure 1b). This results in the transition from 'aerobic' to 'hypoxic' cells occurring about halfway through the viable rim of the spheroid. Addition of 3 mg ml$^{-1}$ of WR-2721 1 hour before irradiation increased the survival of the cells at all positions within the spheroid; the greatest differential in survival was, however, in the cells midway through the rim, and very little additional radioprotection was observed in the outermost or innermost cells. Thus, radioprotection by WR-2721 was maximal in those cells which were marginally hypoxic by radiobiological standards. From a mechanistic point of view, this radioprotection indicates that WR-2721 is dephosphorylated (by cellular or serum enzymes) to its thiol form (WR1065: see Purdie *et al.*, 1983), which then serves as an exogenous thiol source (for brevity, WR-2721 is subsequently described as the active, exogenous thiol). Conversely, treatment with 0.1 mM BSO for 24 hours before irradiation (which depletes glutathione content to less than 5% of control levels (Durand, 1984)) resulted in a sensitisation of all cells within the spheroid; again, the maximum differential occurred in those cells which were marginally (radiobiologically) hypoxic.

As indicated by the data in the upper panel of Figure 1, neither agent resulted in toxicity to any of the subpopulations of cells within the spheroid. These data thus confirm previous results showing the radioprotective and sensitising properties of WR-2721 and BSO respectively. While the nature of the profiles in Figure 1b (increasing thiols resulted in a displacement of the curves to the left) would be consistent with an increasing hypoxic fraction resulting from increased thiol levels, these results could also be adequately explained if these treatments had *no effect* on cellular oxygenation, but rather, if only a limited range of oxygen tensions exists over which thiols can effectively compete with oxygen to reverse potential radiation damage.

The latter hypothesis probably owes its longevity to the difficulty in designing an experiment in which it can be tested. Lacking insight as well, we chose to address the issue from the opposite direction, by determining if cellular oxygenation indeed remained constant. With our current interest in development of fluorescent probes for hypoxic cell identification, application of that technology to the question seemed straightforward. AF-2 is particularly attractive to use in conjunction with the Hoechst staining technique, since the dyes are excited and fluoresce at quite different wavelengths. Consequently, individual cells can be assayed *simultaneously* for both position (Hoechst content) and oxygenation status (AF-2 content). An experimental 'matrix' was thus set up, in which AF-2 uptake in spheroids was measured as a function of cell position, incubation time, WR-2721 concentration and ambient oxygenation. Five concentrations of WR-2721 were used (0.0, 0.5, 1.0, 1.5, 2.0 and 3.0 mg ml$^{-1}$; samples were analysed every 15 minutes for up to 3 hours, all under ambient oxygen atmospheres of 0.08, 0.5, 1, 2, 5 and 10% oxygen. Selected data from these experiments are summarised in the subsequent two figures.

**Figure 1** Clonogenicity of cells recovered from indicated depths within 680 μm V79 spheroids under a 10% oxygen atmosphere. **a** (OGy) shows that the WR-2721 or BSO treatments did not change cellular viability; in **b**, net survival after 15 Gy was increased when WR-2721 was added, and decreased when glutathione was depleted by BSO. The major effect of the thiol manipulations was to change the apparent hypoxic fraction (the curves shifted left/right, rather than up/down). Horizontal lines indicate average survival; 95% confidence limits are shown where they exceeded the size of the plotting symbols. □, 3 mg ml$^{-1}$ WR-2721; △, 0.1 mM BSO; ○, control.

Figure 2 indicates the dependence of spheroid oxygenation (AF-2 binding) on the cellular position in the spheroid (sort fraction, where fraction 1 is the outermost cells), and exposure time to AF-2 and WR-2721. The three-dimensional surfaces in Figure 2 were generated by determining the best-fit linear regression line for AF-2 uptake as a function of exposure time to WR-2721 for each sort fraction, and then interpolating between sort fractions using a polynomial fitting routine. Under 10% oxygen (top panels), little binding of AF-2 was observed in the absence of WR-2721, and in the presence of the radioprotector, binding was seen only in the higher numbered fractions (inner regions of the spheroids). These results are consistent with the known oxygen dependence of AF-2 binding in Chinese hamster V79 cells, with a half maximum value at less than 1,000 parts per million oxygen (i.e. significant binding occurs only at very low oxygen tensions; Olive, 1985).

Consistent with these results, much greater binding was seen when spheroids were incubated in 5% oxygen (middle panels, Figure 2). In this case, both increasing exposure time and increasing depth within the spheroid resulted in greater binding (more hypoxia). Additionally, greater binding of the hypoxic cell probe was observed in the presence of WR-2721 (right panels) than in its absence (left panels). Under more extreme oxygen deprivation conditions, considerably more AF-2 binding was observed (bottom panels). In those lower panels, however, a somewhat different oxygenation profile from the outside to the inside of the spheroids was observed: in the presence of the radioprotector, a more rapid increase in binding with increasing depth into the spheroid was observed. At very low oxygen tensions and for the innermost fractions of cells in the spheroids (those most hypoxic), somewhat less total binding of AF-2 was observed in the presence of the WR-2721 (right panels) than in its absence. This is again consistent with the observed decreases in AF-2 binding previously reported when exogenous thiols were added (Olive, 1981).

Figure 3 presents an additional subset of the data in a different format, where selected populations of cells were analysed (fraction 1 includes the outermost 10% of the cells, fractions 4 and 7 were from more internal regions of the spheroids, and fraction 10 represents the innermost 10% of the cells). The degree of hypoxia (AF-2 binding) in each cell subpopulation is displayed as a function of ambient oxygenation and WR-2721 concentration, all for a 1-hour exposure. The outermost cells of the spheroids, fraction 1 (Figure 3, top panel), responded as expected; little binding was observed until very low oxygen tensions were reached, which then produced a rapid increase in binding rate. Interestingly, at low oxygen levels in these cells, the competition between increased binding by thiol-induced oxygen removal, and reduced binding due to extracellular thiol and hypoxic probe interactions, was observed as an initial increase in binding with low to moderate concentrations of the WR-2721, but with the extracellular competition dominating at the higher WR-2721 concentrations.

These responses were seen in a more exaggerated form in fraction 4, about one-third of the way into the viable rim of cells (Figure 3). Again, little binding was seen at high oxygen tensions; at lower oxygen tensions, binding increased rapidly, and was stimulated to a greater extent by WR-2721 at intermediate oxygen tensions than at very low oxygen levels. Cells from fraction 7, about two-thirds of the way into the spheroid, appear to show the competition between thiol-induced hypoxia and thiol inhibition of AF-2 binding most clearly. In these cells, the addition of WR-2721 markedly enhanced AF-2 binding (thus indicating increased hypoxia) even at the highest oxygen tensions shown (10%). At lower oxygen tensions, however, adding WR-2721 decreased AF-2 binding. Thus, the relative importance of the two processes can be easily appreciated; in the case of intermediate levels of hypoxia, producing additional hypoxia dominated the response. At sufficiently low oxygen tensions, however, AF-2 binding was inhibited by the exogenous thiols. Similar results were seen for the innermost cells (fraction 10), though, as expected, the changes were somewhat less dramatic in the more severely oxygen-deprived cells.

An additional factor indicated by these data is the necessity of nitroreduction for AF-2 binding. Binding of the hypoxic cell probe is not, per se, an indication of oxygenation. It is, rather, dependent upon the 'competition' between the reductive capacity of the cell populations, and the auto-oxidation of the reduced species by ambient oxygen (Olive & Chaplin, 1986). Thus, metabolic activity of the cells is required, and the resulting nitroreduction is reversible by available oxygen. Consequently, the greater AF-2 binding seen in cells from fraction 7 compared to that for cells from fraction 10 suggests comparable levels of hypoxia (at least at low external oxygen tensions), but increased

**Figure 2** Flow cytometry analysis of AF-2 binding in V79 spheroids incubated under the indicated atmospheres in the presence or absence of WR-2721. AF-2 binding is shown as a correlated function of position in the spheroid (fraction 1 is the outermost 10% of the cells) and incubation time for each atmosphere. Note that in all cases binding proceeded linearly with time; WR-2721 produced a maximal effect in cells at intermediate oxygen tensions. Spheroids in the range of 550–750 μm diameter were used in replicate studies; data were thus averaged in terms of sort fraction number rather than depth within the spheroids.

**Figure 3** Flow cytometry analysis of AF-2 binding after 1-hour exposure in selected cell subpopulations from V79 spheroids (fraction 1 again represents the outermost 10% of the cells). Hypoxia is shown as a correlated function of oxygen concentration and WR-2721 level. Note that the dependence of AF-2 binding on oxygenation varied by more than 20-fold (from the minimum at 10% oxygen in the outermost cells, to a maximum in cells of fraction 7 under near-anoxic conditions). Conversely, the maximum inhibition of AF-2 binding by WR-2721 (observed in the external cells, fraction 1, at the lowest oxygen tension) was less than a 2-fold differential.

binding in fraction 7 due to the increased nitroreductive capacity of that cell population.

Direct visualisation of the extent and distribution of hypoxia in spheroids under conditions of similar thiol manipulations is presented in Figure 4. The images show the observed fluorescence after a 1 hour exposure to AF-2 in control spheroids (centre panel), and spheroids treated with 0.1 mM BSO for 24 hours (Figure 4a) or with 2 mg ml$^{-1}$ WR-2721 for 1 hour (Figure 4c). Clearly, the addition of WR-2721 (Figure 4c) resulted in more centrally located binding of AF-2 (increased hypoxia). In the right hand panels, a reversed image is shown, in which those cells displaying an AF-2 fluorescence intensity level great enough to be consistent with radiobiological hypoxia are directly indicated.

Several other features can be extracted from the quantitative image analysis techniques used in Figure 4. As can be appreciated from the grey level pictures, the maximum level of AF-2 binding was largely unaffected by the thiol manipulations; only the location of the cells capable of that degree of binding differed. This can be explained only by a change in the number of severely hypoxic cells (size of the hypoxic cell fraction). Interestingly, increases in AF-2 binding as a function of *both* thiol removal (Figure 4a) and thiol addition (through increased hypoxia, Figure 4c) can be deduced. In Figure 4a, though binding was not at a level consistent with radiobiological hypoxia, the somewhat enhanced binding in the peripheral regions of treated spheroids relative to controls can be appreciated by both the slight increase in intensity of binding, and the greater resolution observed (more detailed structure, as opposed to the 'amorphous' uptake in the control). Additionally, as already discussed relative to Figure 3, these central sections of V79 spheroids showed considerable heterogeneity of AF-2 binding, thus indicating that maximal nitroreduction and binding do not necessarily occur in the innermost (presumably most hypoxic) cell populations.

Some suggestion of increased AF-2 binding near the rim of each spheroid is apparent in these images, unlike the data of Figures 2 and 3 obtained with flow techniques. This results from the different excitation/emission wavelengths used by the two instruments. AF-2 bound by external cells shows a relatively broad excitation range, extending from the UV up to green (350–500 nm), but with maximal emission seen in the blue range with UV excitation. Conversely, in internal cells, bound AF-2 (presumably a different metabolite) is still effectively excited by shorter wavelengths, but emits preferentially at longer wavelengths (> 530 nm). With the flow system, laser-excitation is sufficiently powerful that good data can be obtained with 488 nm excitation/550 nm emission, thus selecting for AF-2 bound to hypoxic cells. Conversely, the combination of reduced excitation power and non-optimal emission filters/reflectors in

**Figure 4** Oxygenation patterns in central sections of 480 μm V79 spheroids incubated with 20 μg ml$^{-1}$ AF-2 for 1 hour under 5% oxygen, as recorded by an image processing system. Increasing the net thiols (top to bottom) increased the number of cells binding near-maximal levels of AF-2, but had little effect on that maximal intensity. Reprocessed images are shown on the right, where all cells containing AF-2 levels consistent with radiobiological hypoxia are highlighted (additionally, an electronic mask shows the location of the rim of the spheroid).

the microscope-based image analysis system requires a compromise: the highest quality images are produced by violet light excitation, but the image is somewhat 'biased' by the blue light-emitting species (which shows a distribution pattern suggesting poor penetration into the spheroid when viewed with UV excitation). Thus, the images of Figure 4 show the location of both forms of the AF-2, and, unlike Figures 2 and 3, include the 'contaminating' emissions from the externally bound form of the drug.

### Discussion

The data presented in this manuscript lead to the conclusion that the degree of oxygenation of the spheroid system is highly dependent upon the level of endogenous and exogenous cellular thiols. The data of Figure 1 indicated that the cellular radiosensitivity profile through the spheroids shifted in a manner that would be consistent with an increased hypoxic fraction in the presence of WR-2721, and a decreased hypoxic fraction when endogenous glutathione levels were markedly reduced. Similarly, addition of WR-2721 to spheroids resulted in complex (but consistent) interactions with a hypoxic cell fluorescent probe: at extremes of oxygenation, the binding of the probe was inhibited by the added WR-2721, whereas at intermediate starting oxygen tensions, an increased binding (due to thiol oxidation resulting in decreased net oxygenation (Purdie, 1980; Purdie *et al.*, 1983)) was observed. Direct observation of the pattern of AF-2 binding in control and thiol-manipulated spheroids (Figure 4) also offered visual evidence that the oxygenation pattern of the spheroids was markedly dependent upon thiol levels.

These data would seem to have important implications concerning the continuing debate as to whether thiol manipulation maximally affects the radiosensitivity of cells with low oxygen levels because: (1) SH groups effectively 'compete' with oxygen only over a limited range of oxygen tension, or (2) thiol oxidation reactions alter the available oxygen, and are of increased significance as oxygen availability decreases. If the former mechanism were dominant, it seems necessary that the second should be of minimal importance. However, our data clearly indicate that thiol manipulation *markedly* influences the oxygenation status of cells with low levels of available oxygen, and thus argue that the second mechanism must be of importance.

From a more practical point of view, these results suggest that the major radiobiological consequence of thiol manipulation in multicell systems is likely to be due to the increased or decreased availability of the natural radiosensitiser, oxygen. Data like those presented in Figure 1 can further quantify the relative 'contribution' of the two mechanisms: thiol addition or depletion can result in dose modification factors as large as 1.1 (as measured at the extremes of oxygen tension, in the innermost or outermost cells of the spheroid), whereas the full range of the oxygen effect (a dose modification factor of up to 3.0) can be observed in the marginally hypoxic cells midway into the spheroid, due to the indirect effects of thiol manipultion. The indirect, oxygen-modulating effects of thiol manipulation thus appear to be capable of dominating the effects on radiosensitivity *in vivo* and, further, emphasise the need for care in ensuring that thiol-depleted 'hypoxic' cells are not more radiosensitive only because of less-complete oxygen removal.

This research was supported by USPHS grants CA-37775 and CA-37879. Skilled technical assistance was provided by Denise McDougal and Nancy LePard.

### References

BIAGLOW, J.E. (1983). The role of thiols in cellular response to radiation and drugs. *Radiat. Res.*, **95**, 437.

BRIDGES, B.A. (1969). Sensitization of organisms to radiation by sulphydryl binding agents. *Adv. Radiat. Biol.*, **3**, 123.

BUMP, E.A., YU, N.Y. & BROWN, J.M. (1982). Radiosensitization of hypoxic tumor cells by depletion of intracellular glutathione. *Science*, **217**, 544.

DENEKAMP, J., MICHAEL, B.D., ROJAS, A. & STEWART, F.A. (1981). Thiol radioprotection *in vivo*: the critical role of tissue oxygen concentration. *Br. J. Radiol.*, **52**, 1112.

DENEKAMP, J., MICHAEL, B.D., ROJAS, A. & STEWART, F.A. (1982). Radioprotection of mouse skin by WR-2721; the critical influence of oxygen. *Int. J. Radiat. Oncol. Biol. Phys.*, **8**, 532.

DURAND, R.E. (1982). Use of Hoechst 33342 for cell selection from multicell systems. *J. Histochem. Cytochem.*, **30**, 117.

DURAND, R.E. (1983). Radioprotection by WR-2721 *in vitro* at low oxygen tensions: implications for its mechanisms of action. *Br. J. Cancer*, **47**, 387.

DURAND, R.E. (1984). Roles of thiols in cellular radiosensitivity. *Int. J. Radiat. Oncol. Biol. Phys.*, **10**, 1235.

GRAY, L.H. (1956). A method of oxygen assay applied to a study of the removal of dissolved oxygen by cysteine and cysteamine. In *Progress in Radiobiology*, Mitchell, J.S., Holmes, B.E. & Smith, C.L. (eds) p. 267. Oliver and Boyd: Edinburgh.

MITCHELL, J.B. & RUSSO, A. (1987). The role of glutathione in radiation and drug induced cytotoxicity. *Br. J. Cancer*, **55**, 96.

OLIVE, P.L. (1981). Evidence suggesting that the mechanism for aerobic and hypoxic cytotoxicity of nitroheterocycles is the same. *Int. J. Radiat. Oncol. Biol. Phys.*, **8**, 687.

OLIVE, P.L. (1985). Fluorescent probes for cellular hypoxia: lack of transfer of fluorescence between cells *in vitro*. *Int. J. Radiat. Oncol. Biol. Phys.*, **11**, 1947.

OLIVE, P.L. & CHAPLIN, D.J. (1986). Oxygen and nitroreductase-dependent binding of AF-2 in spheroids and murine tumors. *Int. J. Radiat. Oncol. Biol. Phys.*, **12**, 1247.

OLIVE, P.L. & DURAND, R.E. (1983). Fluorescent nitroheterocycles for identifying hypoxic cells. *Cancer Res.*, **43**, 3276.

PURDIE, J.W. (1980). Dephosphorylation of WR-2721 to WR-1065 *in vitro* and effect of WR-1065 and misonidazole in combination in irradiated cells. In *Radiation Sensitizers – Their Use in the Clinical Management of Cancer*, Brady, L.W. (ed) p. 330. Masson: New York.

PURDIE, J.W., INHABER, E.R., SCHNEIDER, H. & LABELLE, J.L. (1983). Interaction of cultured mammalian cells with WR-2721 and its thiol, WR-1065: implications for mechanisms of radioprotection. *Int. J. Radiat. Biol. Phys.*, **43**, 517.

RUSSO, A., MITCHELL, J.B., FINKELSTEIN, E., DEGRAFF, W.G., SPIRO, I.J. & GAMSON, J. (1985). The effects of cellular glutathione elevation on the oxyen enhancement ratio. *Radiat. Res.*, **103**, 232.

SUTHERLAND, R.M. & DURAND, R.E. (1976). Radiation response of multicell spheroids – an *in vitro* tumour model. *Curr. Topics Radiat. Res. Q.*, **11**, 87.